# Stereochemistry and Mechanism of Enzymatic and Non-Enzymatic Hydrolysis of Benzylic *sec*-Sulfate Esters

**DOI:** 10.1002/ejoc.201402211

**Published:** 2014-05-06

**Authors:** Michael Toesch, Markus Schober, Rolf Breinbauer, Kurt Faber

**Affiliations:** [a] Department of Chemistry, Organic & Bioorganic Chemistry, University of GrazHeinrichstrasse 28, 8010 Graz, Austria; [b] Department of Organic Chemistry, Graz University of TechnologyStremayrgasse 9, 8010 Graz, Austria

**Keywords:** Synthetic methods, Enzyme catalysis, Hydrolysis, Reaction mechanisms, Configuration determination, Hammett constant

## Abstract

The substrate scope of inverting alkylsulfatase Pisa1 was extended towards benzylic *sec*-sulfate esters by suppression of competing non-enzymatic autohydrolysis by addition of dimethyl sulfoxide as co-solvent. Detailed investigation of the mechanism of autohydrolysis in ^18^O-labeled buffer by using an enantiopure *sec*-benzylic sulfate ester as substrate revealed that from the three possible pathways (i) inverting S_N_2-type nucleophilic attack of [OH^–^] at the benzylic carbon represents the major pathway, whereas (ii) S_N_1-type formation of a planar benzylic carbenium ion leading to racemization was a minor event, and (iii) Retaining S_N_2-type nucleophilic attack at sulfur took place at the limits of detection. The data obtained are interpreted by analysis of Hammett constants of *meta* substituents.

## Introduction

Enantioselective hydrolysis of ester and amide bonds catalyzed by lipases, esterases, and proteases represents a landmark in biotransformations.[1] Their (industrial) application was significantly widened by introduction of dynamic resolution concepts that make use of in situ racemization[2] to overcome the 50 %-yield threshold of kinetic resolution. As an alternative, simultaneous (or stepwise) transformation of a pair of substrate enantiomers through stereochemically opposite pathways leads to deracemization.[3] For the latter concepts, hydrolytic enzymes acting through retention or inversion of configuration are a crucial prerequisite.[4] In this context, we recently developed a deracemization protocol for *rac*-*sec*-alcohols through enantio-complementary hydrolysis of their corresponding sulfate monoesters by using a pair of sulfatases acting through stereo-complementary pathways.[5] The key enzymes employed were the retaining aryl sulfatase, PAS, from *Pseudomonas aeruginosa*[6] and the inverting alkyl sulfatase, Pisa1, from *Pseudomonas* sp. DSM 6611.[7] Fortunately, Pisa1 displayed a very broad substrate spectrum encompassing linear and branched *sec*-sulfate esters that bear various functional groups, such as allylic C=C and propargylic C≡C bonds, which are prone to undergo side reactions with transition metal catalysts used in dynamic resolution protocols.[8] In contrast, benzylic sulfate ester **2a** gave poor results with Pisa1, presumably owing to its hydrolytic instability at pH ≈ 8 going alongside competing spontaneous (non-enzymatic) hydrolysis, thereby eroding the *ee* of product **2b**.[8] By aiming to suppress the background hydrolysis by optimization of reaction conditions, we initiated a detailed study on the mechanism of enzymatic and non-enzymatic hydrolysis of *sec*-allylic and benzylic sulfate esters *rac*-**1a**–**8a**. Although aryl and *n*-alkyl sulfates have been thoroughly investigated regarding their stability towards hydrolysis,[9,10] no detailed studies are available on the hydrolysis of *sec*-alkyl sulfate esters. The majority of investigations deal with detergents, such as sodium dodecyl sulfate [11] or related anionic surfactants,[12] which predominantly consist of primary alkyl sulfates, in which the stereochemical consequences of hydrolysis are not an issue. Studies on highly branched neopentyl sulfate reported rearrangement issues.[13]

## Results and Discussion

During our initial studies[8] we attempted to improve incomplete stereoselectivities observed with several allylic, propargylic and benzylic *sec*-sulfate esters by addition of dimethyl sulfoxide (DMSO). Although positive effects were observed, the exact molecular reason for this selectivity-enhancement – suppression of spontaneous (non-enzymatic) hydrolysis and/or alteration of the catalytic properties of the enzyme[14] – remained unknown.

The influence of the polarity of water-miscible organic co-solvents on the *ee* of **1b** obtained from non-enzymatic hydrolysis of enantiopure (*S*)-**1a** was investigated (Table 1). Although significant racemization took place in neat buffer [*ee* of (*R*)-**1b** 34 %, *E*_T_^N^ of H_2_O ≈ 1], this effect gradually diminished upon decreasing the polarity (as indicated by the Dimroth–Reichardt parameter *E*_T_^N^)[15] of the organic co-solvent used [*ee* of (*R*)-**1b** 48 %, *E*_T_^N^ of DMSO 0.44]. Reducing the reaction temperature from 60° to 20 °C in Tris-buffer in the absence of organic co-solvent had a similar effect (*ee*_P_ 25 % versus 52 %, respectively). Both effects indicate the involvement of a polar (e.g. an allylic carbenium ion) species.

**Table 1:** Non-enzymatic hydrolysis of (*S*)-1-octen-3-yl sulfate (1a) in the presence of water-miscible organic co-solvents.[a] 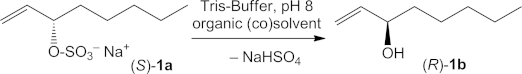

Co-Solvent	*ee* of (*R*)-1b[%]	Dimroth–Reichardt parameter (*E*_T_^N^)[b]
None	34	≈ 1
Methanol	44	0.76
Ethanol	45	0.65
2-Propanol	46	0.55
DMSO	48	0.44

[a] Conditions: Tris-buffer 100 mm, pH 8.0, 20 % (v/v) co-solvent, 5 mg/mL (*S*)-**1a**, 90 h at 30 °C.

[b] *E*_T_^N^ values are given for pure solvents.

**Scheme 1:**
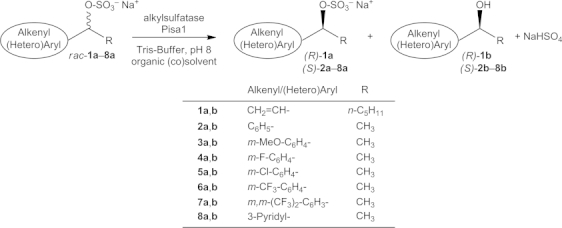
Stereoselective hydrolysis of allylic and benzylic *sec*-sulfate esters by using inverting alkylsulfatase Pisa1.

To support the hypothesis that a polar carbenium ion species causes racemization during non-enzymatic hydrolysis, a series of benzylic *sec*-sulfate esters (**2a**–**8a**) were subjected to non-enzymatic and enzymatic hydrolysis with Pisa1 (Scheme 1, Table 2). Of special interest were the *meta* substituted derivatives **2a**–**6a**, because the electronic effects of the *meta* substituents on the (de)stabilization of a benzylic carbenium ion can be easily correlated to their Hammett constants.[16] Substrate **7a** was incorporated from ref.[8] for comparison and pyridyl-analog **8a** was used as an electron-deficient heterocyclic candidate.

**Table 2:** Enzymatic and non-enzymatic hydrolysis of benzylic *sec*-sulfate esters *rac*-**2a–8a**.

Substrate	Co-solvent[a]	Conversion [%][b]	*ee*_P_ [%]	*E*[c]	Autohydrolysis [%][d]	*σ*_m_ constant[e]
*rac*-**2a**[f]	none	> 99	3.6	< 2	> 96	0.00
	DMSO	> 99	4.1	< 2	> 96	
*rac*-**3a**	none	99	40	2.6	96	0.12
	DMSO	82	82	10	34	
*rac*-**4a**	none	60	60	12	13	0.34
	DMSO	50	93	96	2	
*rac*-**5a**	none	60	61	13	10	0.37
	DMSO	50	93	84	1	
*rac*-**6a**	none	54	85	70	4	0.43
	DMSO	50	99	> 200	0.3	
*rac*-**7a**[g]	none	10	> 99	> 200	< 0.3	n.a.
	DMSO	13	> 99	> 200	< 0.3	
*rac*-**8a**	none	50	99	> 200	12	n.a.
	DMSO	48	> 99	> 200	6	

[a] Standard conditions: Pisa1 (0.13 mg), Tris-buffer, 100 mM, pH 8.0, substrate **2a**–**8a** (5 mg/mL), 24 h at 30 °C; ^a^ 20 % v/v.

[b] Calculated from *ee*_S_/(*ee*_S_ + *ee*_P_).

[c] Enantiomeric Ratio (*E*) calculated from *ee*_P_ and *ee*_S_: *E* = {ln[(1-*ee*_S_)/(1 + *ee*_S_/*ee*_P_)]}/{ln[(1 + *ee*_S_)/(1 + *ee*_S_/*ee*_P_)]};[17] for the application of *E* values to kinetic resolutions with competing autohydrolysis, see ref.[18]

[d] Conversion in the absence of enzyme.

[e] Hammett constant of substituent R in the *meta* position (Scheme 1).

[f] For data from 6 h reaction time, see ref.[8].

[g] For data from 72 h reaction time, see ref.[8]; n.a. = not applicable.

Substrates *rac*-**2a**–**8a** were subjected to enzymatic hydrolysis under standardized reaction conditions by using Pisa1, the *ee*_P_ of *sec*-alcohols (*S*)-**2b**–**8b** formed was determined by GC analysis on a chiral stationary phase after extractive separation from the remaining non-hydrolyzed sulfate esters (*S*)-**2a**–**8a**. The latter were subjected to acid-catalyzed hydrolysis through strict retention of configuration[8] to yield corresponding alcohols (*S*)-**2b**–**8b** for *ee*-determination. Autohydrolysis was measured under identical conditions in the absence of enzyme. Absolute configurations were elucidated by co-injection with authentic reference materials with known absolute configuration.[8] DMSO was selected as co-solvent because it showed the strongest selectivity-enhancing effects (Table 1).

The enzymatic hydrolysis of substrate *rac*-**2a** was strongly outcompeted by non-selective autohydrolysis and consequently gave alcohol **2b** in near racemic form. Although the addition of DMSO showed a positive trend, the effects were too small to be truly beneficial.

Introduction of electron-withdrawing substituents in the *meta* position (substrates **3a**–**6a**) gave increasingly better results, i.e. the gradual suppression of autohydrolysis gave a strong improvement in the apparent enantioselectivities[18] from barely detectable (*E* = 2.6) to a respectable value (*E* = 70). In line with the suppression of autohydrolysis, the overall reaction rates slowed from **2a** to **6a**, indicated by decreasing conversion values. The addition of DMSO (20 % v/v) further decreased autohydrolysis and hence gave even better overall enantioselectivities of up to *E* > 200. The correlation between the electronic properties of the *meta* substituents, as denoted by their Hammett *σ*_m_-values, is remarkably strong: there is a drastic improvement in selectivity owing to decreased autohydrolysis going from R = H (0.00) through R = MeO (0.1) to R = Hal (> 0.3), whereas both halo-derivatives with comparable *σ*_m_-values of 0.34 and 0.37 gave similar results. A further significant improvement was achieved with the CF_3_-derivative (0.43).

The beneficial effect of electron-deficient substituents in the *meta* position is nicely underlined by doubly *meta* substituted substrate **7a**, which could be resolved with perfect enantioselectivity.[8] To test whether this electronic effect could also be extended to heteroaromatic benzylic analogs, electron-deficient 3-pyridyl derivative **8a** was investigated. In line with the above trends, it could be resolved with excellent results (*E* > 200). Unfortunately, attempts to synthetize electron-rich derivatives, such as 1-(furan-2yl)ethyl sulfate, 1-(thiophen-2-yl)ethyl sulfate, 1-(1*H-*pyrrol-2-yl)ethyl sulfate or imidazole analogs, which could serve as counterproof, were unsuccessful owing to the instability of the corresponding *sec*-alcohols.

The hydrolysis of *sec*-alkyl monosulfate esters is a complex process: Acid catalysis proceeds by protonation of the negatively charged sulfate ester moiety[19] at the C–O–S bridge atom, which allows nucleophilic attack of H_2_O at sulfur, along with release of the alcohol and HSO_4_^–^ as a good leaving group.[20] Consequently, it is a fast process and proceeds with retention of configuration at the chiral C-atom bearing the sulfate ester moiety. However, nucleophilic attack of [OH^–^] at C under basic conditions would proceed through inversion at C, but it is hardly possible, because the approach of [OH^–^] onto the negatively charged substrate is disfavored and the process would generate SO_4_^2–^ as a poor leaving group; hence, it is an exceedingly slow process.[21,22] In contrast, the enzymatic hydrolysis as exemplified by inverting alkylsulfatase Pisa1 is a masterpiece of cooperative acid-base catalysis:[7] Nucleophilic attack of [OH^–^] onto C (derived from H_2_O by a binuclear Zn^2+^ cluster in the active site of the enzyme) is complemented by simultaneous protonation of the sulfate ester moiety through histidine 317 to generate HSO_4_^–^. All of these processes basically proceed through S_N_2 at C, because the generation of an aliphatic carbenium ion would be energetically too costly.

**Scheme 2:**
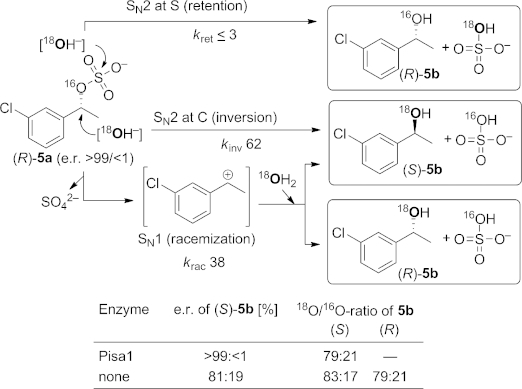
Elucidation of retaining (S_N_2 at S), inverting (S_N_2 at C) and racemizing (S_N_1) pathways of non-enzymatic and enzymatic hydrolysis of (*R*)-5a through ^18^O-labeling (*k* values are stated as first order relative rate constants).

With benzylic substrates, such as **5a**, a resonance-stabilized carbenium ion has to be taken into account, because clear correlation of the decrease of autohydrolysis with the electron-withdrawing effects of *meta* substituents (as indicated by their Hammett constants) strongly suggests that autohydrolysis (at least in part) occurs through an S_N_1-mechanism via an intermediate benzylic carbenium ion. Our investigations on the mechanism of autohydrolysis was led by the following considerations: (i) analysis of the *ee* of formed alcohol **5b** (and its potential erosion) derived from enantiopure substrate (*R*)-**5a** would prove the existence of a transient benzylic carbenium ion responsible for racemization; (ii) use of ^18^O-labelled water would allow determination of the site of nucleophilic attack (S versus C) through incorporation of [OH^–^] either into the formed alcohol (attack at C) or into inorganic sulfate (attack at S) to prove inversion or retention of configuration, respectively (Scheme 2).

To check the validity of the method, enzymatic hydrolysis of (*R*)-**5a** [enantiomer ratio (e.r.) >99:<1] by using inverting Pisa1 in ^18^OH_2_ was performed as a control experiment.[7] For handling purposes, the medium was composed of ^16^O-Tris-buffer (0.1 mL, 1 M, pH 8.0) diluted at a ratio of 1:10 with ^18^O-labelled H_2_O (label 97:3). Addition of Pisa1 (2.6 mg) from 4.6 μL of ^16^OH_2_ stock solution led to a (calculated) ^18/16^O-ratio in the reaction medium of 84:16. After 24 h of reaction time, analysis of alcohol **5b** by GC–MS with a chiral stationary phase revealed an e.r. of >99:<1 for the (*S*)-enantiomer with an ^18/16^O-label of 79:21. These data confirm that Pisa1 hydrolyzed (*R*)-**5a** with complete inversion with concomitant incorporation of ^18^O at C within the limits of accuracy (calculated 84:16, measured 79:21).

The pathways of autohydrolysis were investigated by an analogous experiment in the absence of enzyme by using an ^18/16^O-label of 83:17 at a fivefold-extended reaction time. The following facts were deduced:

(i) Non-enzymatic hydrolysis of enantiopure (*R*)-**5a** (e.r. >99/<1) gave (*S*)-**5b** with an e.r. of 81:19, indicating that inversion through S_N_2 at C is a dominant pathway.

(ii) The (*R*)-enantiomer of alcohol **5b** derived from (*R*)-**5a** can either be formed through retention or racemization, but ^18^O-labeling of (*R*)-**5b** can only take place through racemization, because retention retains the ^16^O-label. Because the ratio of ^18/16^O-label in (*R*)-**5b** (79:21) corresponds to that of the aqueous medium (83:17) within the accuracy of measurement, it can be concluded that retention at C through S_N_2 at S can be neglected and racemization through S_N_1 through a benzylic carbenium ion strongly prevails.

(iii) Consequently, inversion (S_N_2 at C) and racemization (S_N_1) are the major pathways. Their relative proportion can be estimated by taking the erosion of e.r. from (*R*)-**5a** to (*S*)-**5b** (e.r. from >99*R*:<1*S* to 81*S*:19*R*) into account: Because racemization produces equal amounts of (*R*)- and (*S*)-**5b** (19 parts each, i.e. 38 in total), the remainder of 62 parts counts for inversion (considering retention below the limits of detectability ≤ 3). Consequently, the ratio of relative rates of *k*_inv_ (S_N_2 at C) versus *k*_rac_ (S_N_1) are about 1.6:1.

## Conclusions

The enantioselectivity of the enzymatic hydrolysis of benzylic *sec*-sulfate esters by using inverting alkylsulfatase Pisa1 could be significantly improved by suppressing the autohydrolysis of substrates by addition of DMSO as co-solvent. H_2_^18^O-Labeling studies revealed that the major pathway of autohydrolysis proceeded through S_N_2-type inversion at carbon. In contrast, nucleophilic attack at sulfur and the S_N_1-type pathway through a benzylic carbenium ion took place at the limits of detection. The data obtained are interpreted by analysis of Hammett constants of *meta* substituents. These results contribute to the understanding of the bioactivity of sulfated steroids possessing carcinogenic[23] or anabolic properties[24] and the stereo-complementary nucleophilic substitution of sulfur-based leaving groups.[25]

## Experimental Section

**Enzymatic Hydrolysis of Sulfate Esters 3a–6a and 8a:** The corresponding sulfate ester **3a**–**6a** and **8a** (5 mg) was dissolved in Tris/HCl buffer (1 mL, 100 mM, pH 8.0), Pisa1 was added (0.13 mg) and the reaction was shaken with 120 rpm for 24 h at 30 °C. Afterwards, ethyl acetate (1 mL) was added and the mixture was centrifuged for 3 min at 13.000 rpm. The organic phase was separated and dried with Na_2_SO_4_ and alcohols **3b**–**6b** and **8b** were derivatized to the corresponding acetates with DMAP (1 mg) and acetic anhydride (100 μL) overnight. The reaction was quenched by addition of H_2_O (300 μL) with stirring for 3 h. After centrifugation for 3 min at 13.000 rpm, the organic phase was dried with Na_2_SO_4_ and directly measured with GC-FID. The enzymatic hydrolysis of substrates **1a**, **2a** and **7a** is described elsewhere.[8]

**Quantification of Autohydrolysis:** The respective sulfate ester **3a**–**6a** and **8a** was dissolved in Tris/HCl-buffer (1 mL, 100 mM, pH 8.0) and were shaken at 120 °C and 30 rpm for 24 h. The reaction was quenched by freezing in liquid N_2_ and was thawed individually prior to measurement. Quantification of autohydrolysis was done from calibration curves with the corresponding alcohol and sulfate ester.

All measurements were carried out with a Shimadzu HPLC system (CBM-20A, LC-20AD, DGU-20A5, SIL-20AC, CTO-20AC, SPD-M20A, CBM-20A) by using a ZORBAX 300-SCX (4.6 × 250 mm) IEX column and UV-detection [diode array detector set at 271 nm (**3a**), 261 nm (**4a**), 266 nm (**5a**), 262 nm (**6a**) and 259 nm (**8a**)]. The conversion was determined by using sodium formate buffer (200 mM pH 2.8) at a flow rate of 0.5 mL/min and a run time of 20 min (for retention times see Supporting Information, Table S1).

**^18^O-Labeling Experiments:** ^18^O-Enriched water (90 μL, ^18^O content 97 %) was added to a buffer solution (^16^OH_2_ 10 μL, 1 M Tris/HCl pH 8.0) to reach a final buffer concentration of 100 mM (^18/16^O-label 83:17). Substrate (*R*)-**5a** (1 mg) was added to the solution and was shaken for 24 h at 30 °C and 120 rpm. Afterwards, alcohol **5b** was extracted with ethyl acetate (0.1 mL), the organic phase was dried with Na_2_SO_4_ and directly measured with GC–MS. GC–MS measurements were carried out with an Agilent 5975C MS connected to an Agilent 7890A GC fitted with a CTC Analytics PAL Autosampler by using a Chirasil Dex CB column (25 m × 0.32 mm × 0.25 μm film) and He as a carrier gas (0.69 bar). Injection temperature 250 °C, flow 0.5 mL/min, temperature program: 80° hold 1 min, 15 °C/min to 141 °C, 0.5 °C/min to 143 °C, 17 °C/min to 180 °C. Retention times: (*R*)-**5b** 7.4 min, (*S*)-**5b** 7.7 min.

Enzymatic reactions were performed analogously to the control reaction with addition of Pisa1 (26 μg, 353 pmol, 4.6 μL of stock solution in ^16^OH_2_).

**Supporting Information** (see footnote on the first page of this article): Expression of PISA1, synthesis of substrates and reference compounds, analytical methods, NMR and MS spectra, and optical rotation values are presented.

## Supporting Information

(see footnote on the first page of this article): Expression of PISA1, synthesis of substrates and reference compounds, analytical methods, NMR and MS spectra, and optical rotation values are presented.
