# Supplementary material for: Stereochemistry and Mechanism of Enzymatic and Non-Enzymatic Hydrolysis of Benzylic sec-Sulfate Esters
Source: European J Org Chem. 2014 May 6;2014(18):3930–4. doi: 10.1002/ejoc.201402211 (PMC4163651; doi:10.1002/ejoc.201402211)
Supplement: Supplementary file 1 [file ejoc2014-3930-sd1.pdf]

Eur. J. Org. Chem. **2014** · © WILEY-VCH Verlag GmbH & Co. KGaA, 69451 Weinheim, 2014 · ISSN 1099–0690

SUPPORTING INFORMATION

DOI: 10.1002/ejoc.201402211
Title: Stereochemistry and Mechanism of Enzymatic and Non-Enzymatic Hydrolysis of Benzylic *sec*-Sulfate Esters
Author(s): Michael Toesch, Markus Schober, Rolf Breinbauer, Kurt Faber*

General

Rac-1-(3-fluorophenyl)ethanol **4b** and *rac*-1-(3-chlorophenyl)ethanol **5b** were purchased from Alfa Aesar, *m*-methoxyacetophenone **3c**, *m*-fluoroacetophenone **4c**, *m*-chloroacetophenone **5c**, 1-[3-(trifluoromethyl)phenyl]ethanone **6c**, 3-acetylpyridine **8c**, *rac*-1-octen-3-ol **1a** and (*S*)-1-octen-3-ol (*S*)-**1a** were obtained from Sigma Aldrich and (*R*)-1-octen-3-ol (*R*)-**1a** was purchased from Acros. ^{18}O-Labeled water was obtained from Euriso-top (label 97 %). ADH-A from *Rhodococcus ruber* was prepared as reported.[1] ADH Evo 1.1.200 was purchased from Evocatal. Expression and purification of sulfatase Pisa1 from *Pseudomonas* sp. DSM 6611 was performed as reported.[2] Flash chromatography was performed on Merck silica gel 60 (0.040-0.063 mm). TLC was performed using precoated aluminum sheets with silica gel 60. Alcohol products were visualized using UV (245 nm) and/or staining with a Ce-Mo staining solution.

Synthesis of substrates and reference compounds

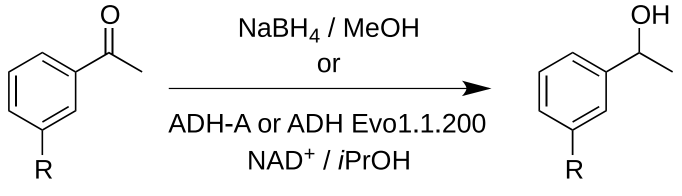

3c-6c, 8c

rac-**3b**, *rac*-**6b,** *rac*-**8b**
(*S*)-**3a-6a**,(*S*)-**8a**, (*R*)-**4a**

Scheme S1: Reduction of ketones **3c-6c** and **8c** using $NaBH_4$ to yield *rac*-**3b**, *rac*-**6b** and *rac*-**8b** or asymmetric bioreduction using ADH-A or ADH Evo 1.1.200 to obtain (*S*)-**3a-6a**, (*S*)-**8a** and (*R*)-**4a**.

Rac-1-(3-methoxyphenyl)ethanol **3b**, *rac*-1-[3-(trifluormethyl)phenyl]ethanol **6b**, *rac*-1-(pyridine-3-yl)ethanol **8b**:

In a 100 mL round bottom flask equipped with a magnetic stirring bar were put: 8 mM of the corresponding ketone (**3c**, **6c**, **8c**), 12 mM of $NaBH_4$ and 30 mL of MeOH. The reactions were

stirred at r.t. for 2 hours or until no more starting material was visible on TLC. The reaction mixture was evaporated, washed with saturated NaCl and dried with Na_2SO_4. Products *rac*-**3b**, *rac*-**6b** and *rac*-**8b** were purified by flash chromatography using the following eluents: *rac*-**3b**: Ethyl acetate/hexane (4/6); *rac*-**6b**: ethyl acetate/hexane (1/5); *rac*-**8b**: ethyl acetate.

Asymmetric bioreduction of ketones **3c-6c** and **8c** using ADH-A to obtain (*S*)-**3b-6b** and (*S*)-**8b:**

In a 50 mL flask were put NAD^+ (2 mg), ketone **3c-6c** (100 mg), *i*-propanol (2 mL), ADH-A (40 U from a 1 mL stock solution) and phosphate-buffer (10 mL, 50 mM, pH 7.5). The reaction was carried out at 30 °C with shaking at 120 rpm for 72 h. Afterwards, the water phase was extracted with ethyl acetate (3 x 10 mL), dried with Na_2SO_4 and the solvent was evaporated. The resulting oil was subjected to flash chromatography with following eluents: (*S*)-**3b**, (*S*)-**6b** and (*S*)-**8b** were eluted as their racemic counterparts, (*S*)-**4b** and (*S*)-**5b**: ethyl acetate/hexane (1/5).

Asymmetric reduction of **4c** using ADH Evo 1.1.200 for the synthesis of (*R*)-**4b**:

In a 50 mL flask were put NAD^+ (30 mg), ketone **4c** (200 mg), *i*-propanol (3 mL), ADH Evo 1.1.200 (50 mg) and of phosphate-buffer (27 mL, 50 mM, pH 6.5). The reaction was carried out at 30 °C with shaking at 120 rpm for 24 h. Afterwards, the water phase was extracted ethyl acetate (3 x 10 mL), dried with Na_2SO_4 and the solvent was evaporated. The resulting oil was subjected to flash chromatography using ethyl acetate/hexane (1/5) as eluent.

Preparation of sulfate esters

Substrates *rac*-**1a-8a**, (*S*)-**1a** and (*R*)-**5a**, were prepared following a known procedure with the following modification:[2] NaH (60% suspension in mineral oil, Sigma Aldrich) was added directly in 1.2 molar excess. Isolated yields were as follows: *rac*-**3a**: 47 %; *rac*-**4a**: 67 %; *rac*-**5a**: 60 %; (*S*)-**5a**: 67 %; *rac*-**6a**: 77 %; *rac*-**8a**: 69 %. Yields for *rac*-**1a**, (*S*)-**1a** and *rac*-**2a** are reported elsewhere.[3]

Stereoselectivities of Pisa1 were determined according to a known procedure.[2]

Determination of absolute configuration

The absolute configuration of alcohols **1a-8a** was determined via co-injection with authentic reference material on GC. Reference material (*S*)-**3b-6b** and (*S*)-**8b** was obtained by asymmetric bioreduction of the corresponding ketones **3c-6c** and **8c** employing (*S*)-selective ADH-A as previously described. The absolute configuration of (*S*)-**3b-6b** and (*S*)-**8b** was determined by optical rotation. $[\alpha]_D^{20}$-Values were measured at 20 °C on a Perkin–Elmer Polarimeter 341 using the sodium D-line. (*S*)-**3b**: $[\alpha]^D_{20}$ -39.4° (c 1.08, $CHCl_3$), lit:[4] -42.1° (c 1.0, $CHCl_3$ (*S*)-**4b** $[\alpha]^D_{20}$ -41.0° (c 1.03, $CHCl_3$), lit:[5] -24.4° (c 1.24, $CHCl_3$); (*S*)-**5b**: $[\alpha]^D_{20}$ -38.0° (c 1.11, $CHCl_3$), lit:[6] -41.0° (c 1.41, $CHCl_3$); (*S*)-**6b** $[\alpha]^D_{20}$ -33.8° (c 1.0, $CHCl_3$), lit:[7] -31.0° (c 1.95, $CHCl_3$); (*S*)-**8b**: $[\alpha]^D_{20}$ -55,9°

(c 1.0, $CHCl_3$), lit:[8] -49.0° (c 0.84, $CHCl_3$). The absolute configuration of (*R*)-**5b** was determined via GC-FID using *rac*- and (*S*)-**5b** as standard.

Table S1. Retention times for sulfate esters **3a-6a** and **8a** and alcohols **3b-6b** and **8b** on HPLC.

Compound	Retention time [min]	
	Sulfate	Alcohol
**3a,b**	**3a** 6.2	**3b** 9.3
**4a,b**	**4a** 6.3	**4b** 8.5
**5a,b**	**5a** 6.6	**5b** 9.5
**6a,b**	**6a** 6.1	**6b** 10.1
**8a,b**	**8a** 7.2	**8b** 10.2

Autohydrolysis experiments with enantiopure (*S*)-1-Octen-3-yl sulfate

Temperature study: Sulfate ester (*S*)-**1a** (5 mg) was dissolved in buffer (1 mL, 100 mM Tris/HCl pH 8.0) and was shaken in an Eppendorf thermomixer comfort at 20, 30, 40, 50 and 60 °C, respectively at 450 rpm for 6 and 24 h. Afterwards, the product alcohol was extracted with ethyl acetate (1 mL), dried with Na_2SO_4 and derivatised by acetylation as described before and measured on GC-FID for e.e.-determination.

Cosolvent study: (*S*)-**1a** (5 mg) was dissolved in Tris/HCl-buffer (0.8 mL, 100 mM, pH 8.0) and 0.2 mL of the respective cosolvent was added to the solution. The reaction was carried out for 90 h at 30 °C and 120 rpm. Afterwards, the alcohol was extracted with 1 mL of ethyl acetate, dried with Na_2SO_4 and derivatised as described before and measured on GC-FID for e.e.-determination.

Determination of conversion and enantiomeric exccess

GC-FID analyses were carried out on an Agilent 7890A with FID detector using He as a carrier gas (0.56 bar) and a Chirasil Dex CB column (25 m x 0.32 mm x 0.25 µm film). The conversion was calculated from: conv. [%] = $(e.e._S)/(e.e._S + e.e._P)*100$. All $e.e._s$ were determined following a known procedure via a two step protocol.[2] All substrates **1b-8b** were measured as acetates with the following method: Injector temperature 200 °C, flow 2.0 mL/min He; temperature program: 80 °C, hold for 1.0 min, 15 °C/min to 140 °C, 4 °C/min to 160 °C, 10 °C/min to 180 °C. Determination of the e.e. of **1a**, **2a** and **7a** is described elsewhere.[3]

Table S2. Retention times for alcohols **3b-6b** and **8b** on GC after derivatization to acetates.

Compound	Retention time [min]	
(acetate ester)	(*R*)	(*S*)

**3b**	8.2	8.0
**4b**	5.7	5.4
**5b**	7.6	7.4
**6b**	4.9	4.7
**8b**	7.1	6.9

NMR-Spectroscopy

All NMR-spectra were recorded on a Bruker spectrometer at 300 (^{1}H) and 75 (^{13}C) MHz. Shifts (δ) are given in ppm and coupling constants (*J*) are given in Hz.

***rac*-1-(3-Methoxyphenyl)ethyl sulfate (3a)**

^{1}H NMR (300 MHz, DMSO-d$_6$) δ = 1.41 (d, *J* = 6.6, 3H), 3.74 (s, 3H), 5.16 (m, 1H) 6.80 – 6.67 (m, 1H), 6.94 – 6.82 (m, 2H) 7.21 (m, 2H). ^{13}C NMR (75 MHz, DMSO-d$_6$): δ = 23.85. 55.38, 73.77, 112.0, 112.6, 118.5, 129.4, 146.2, 159.5.

***rac*-1-(3-Fluorophenyl)ethyl sulfate (4a)**

^{1}H-NMR (300 MHz, D_2O): δ = 1.49 (d, *J* = 6.6 Hz, 3H), 5.37 (m, 1H), 6.90 - 7.22 (m, 3H), 7.25 - 7.44 (m, 1H). ^{13}C-NMR: (75 MHz, D_2O): δ = 22.48, 77.43, 112.6, 115.9, 114.7, 115.0, 121.7, 127.8, 130.3, 130.4, 144.1, 144.2, 160.9, 164.2.

***rac*-1-(3-Chlorophenyl)ethyl sulfate (5a)**

^{1}H-NMR (300 MHz, D_2O): δ = 1.48 (d, *J* = 6.6 Hz, 3H), 5.31-5.38 (m, 1H), 7.08 – 7.50 (m, 4H). ^{13}C-NMR (75 MHz, D_2O): δ = 22.43, 77.69, 124.3, 125.9, 128.1, 130.1, 133.7, 143.6.

***rac*-1-(3-(Trifluoromethyl)phenyl)ethyl sulfate (6a)**

^{1}H-NMR (300 MHz, D_2O): δ =1.44 (d, *J* = 6.6 Hz, 1H), 5.26-5.33 (m, 1H), 7.52-7.67 (m, 4H). ^{13}C-NMR: (75 MHz, D_2O): 23.83, 73.15, 122.8, 124.0, 128.9, 129.4, 129.5, 130.5, 146.0.

***rac*-1-(Pyridin-3-yl)ethyl sulfate (8a)**

^{1}H-NMR (300 MHz, D_2O): δ = 1.52 (3H, d, *J* = 6.6 Hz), 5.39-5.46 (1H, m), 7.34-7.39 (1H, m) 7.81 (1H, d, *J*=8.0 Hz), 8.36-8.46 (2H, m). ^{13}C-NMR (75 MHz, D_2O): δ = 22.14, 76.03, 124.2, 135.1, 137.6, 146.5, 148.3.

rac-1-(3-Methoxyphenyl)ethyl sulfate (**3a**) DMSO-d_6

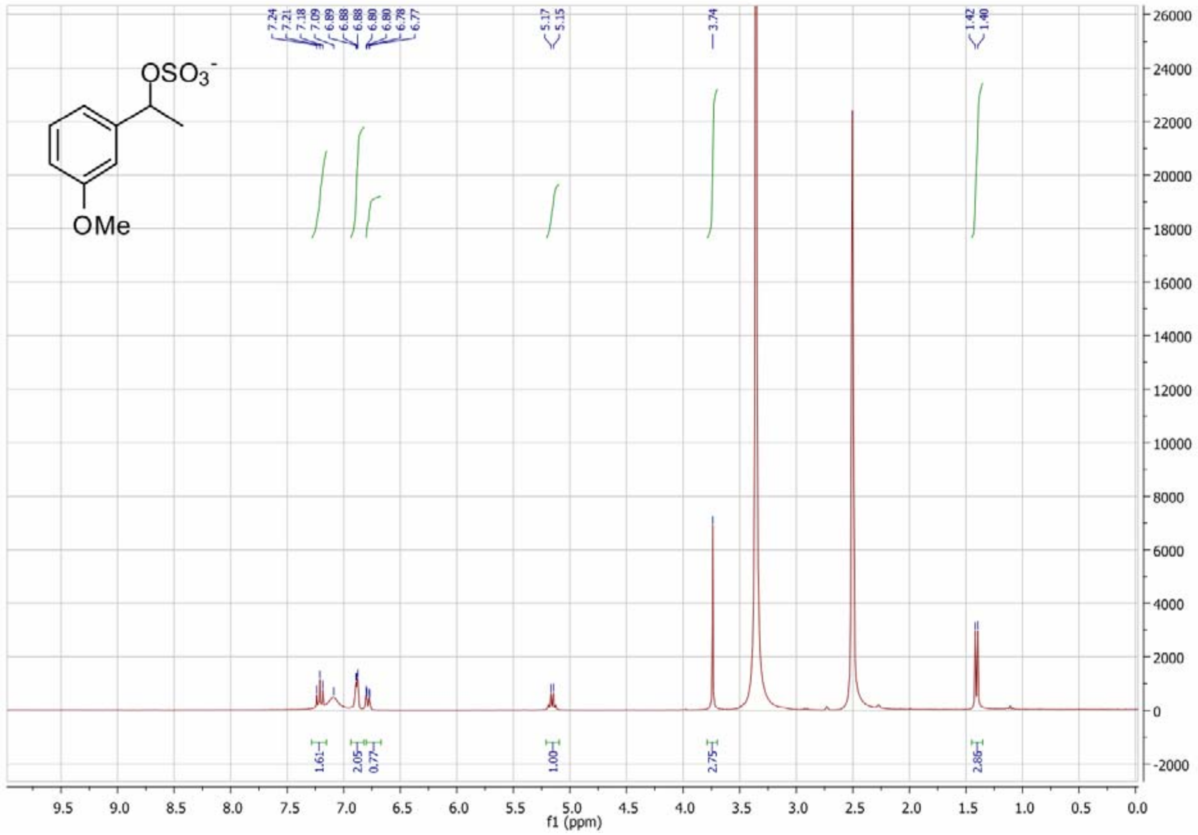

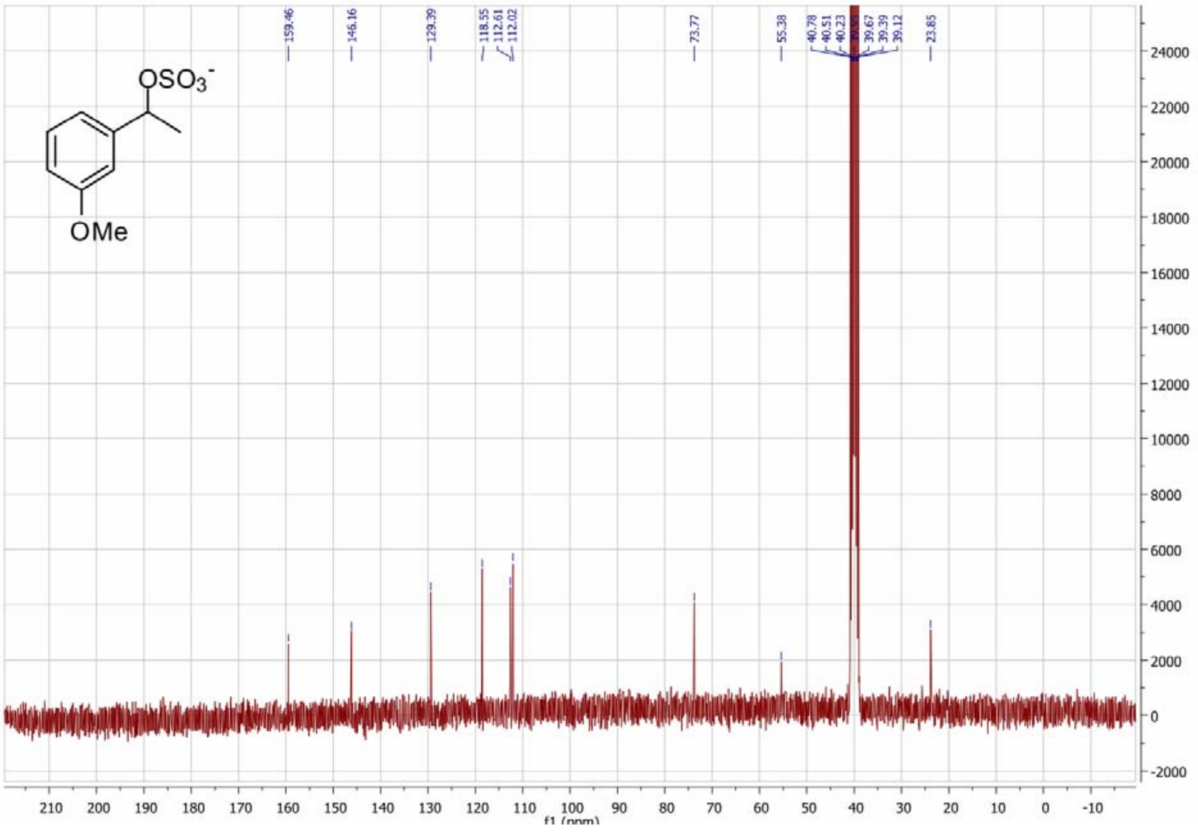

rac-1-(3-Fluorophenyl)ethyl sulfate (**4a**) D_2O

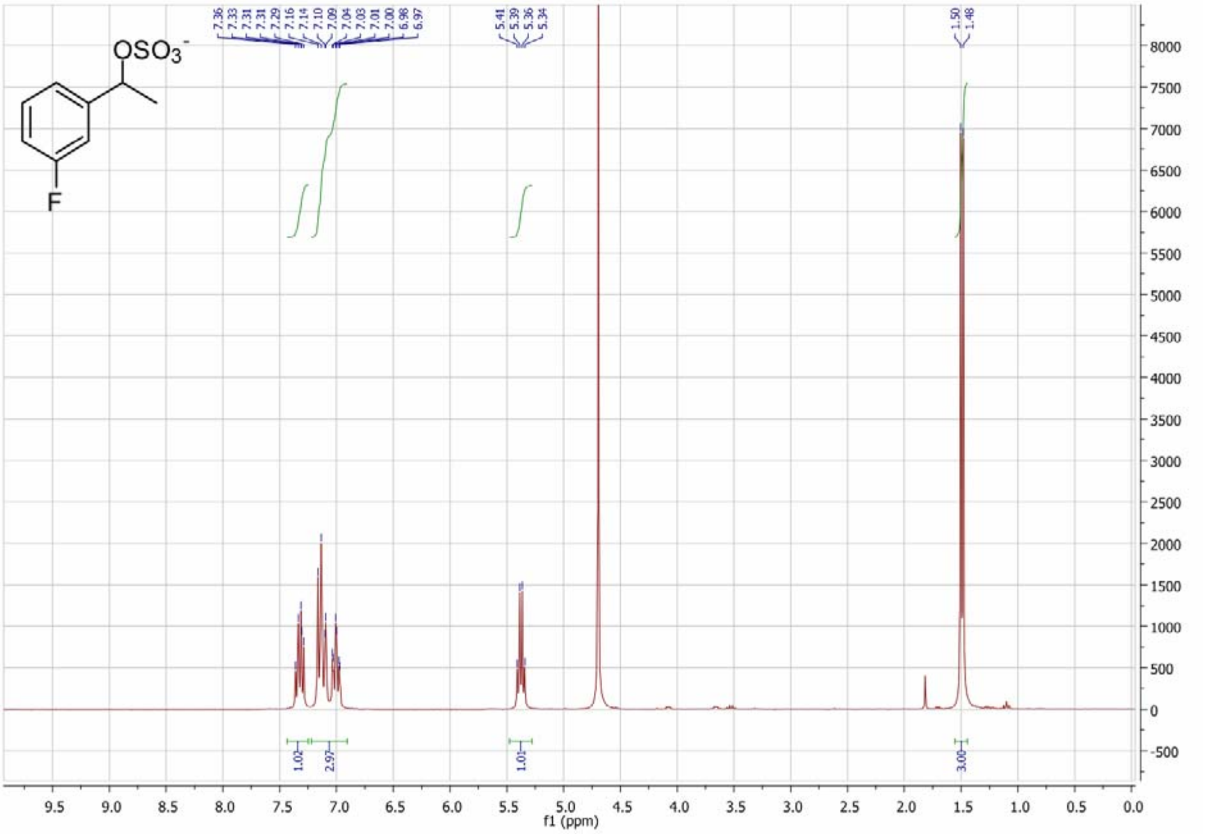

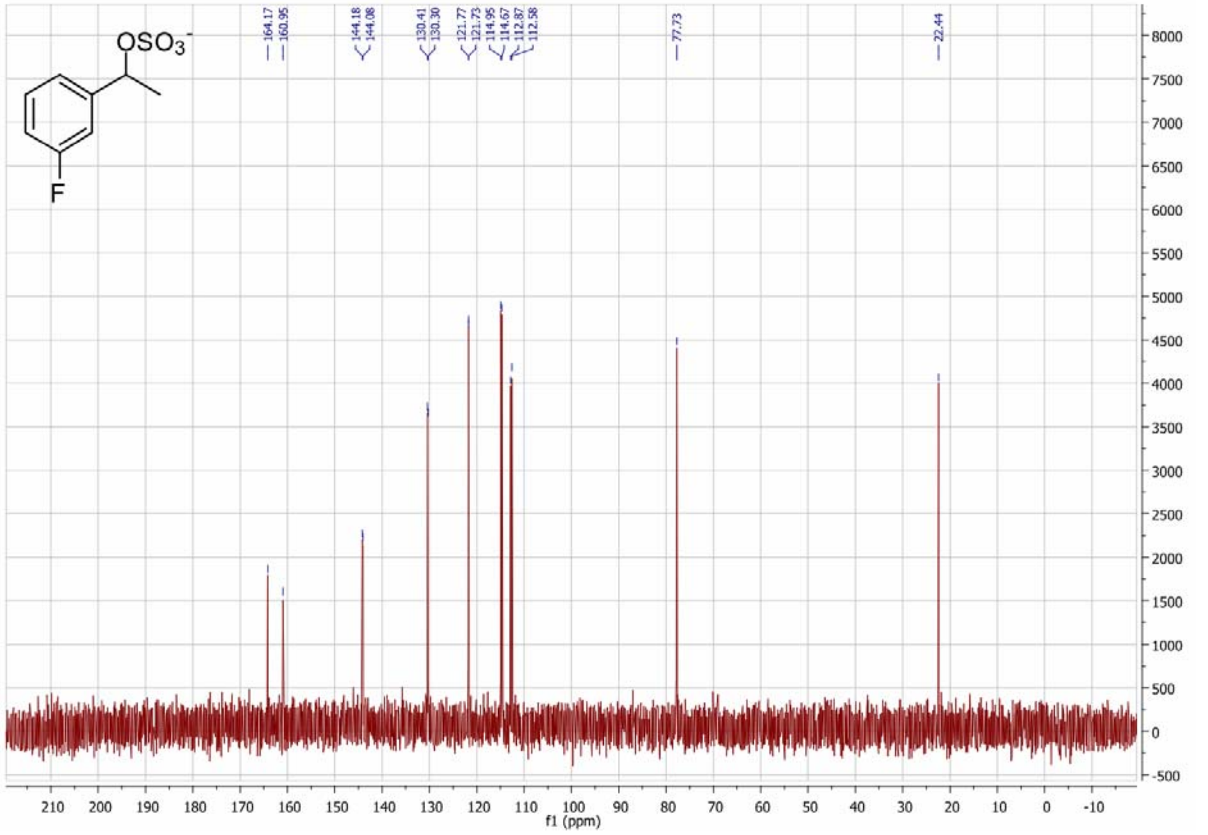

rac-1-(3-Chlorophenyl)ethyl sulfate (**5a**) D_2O

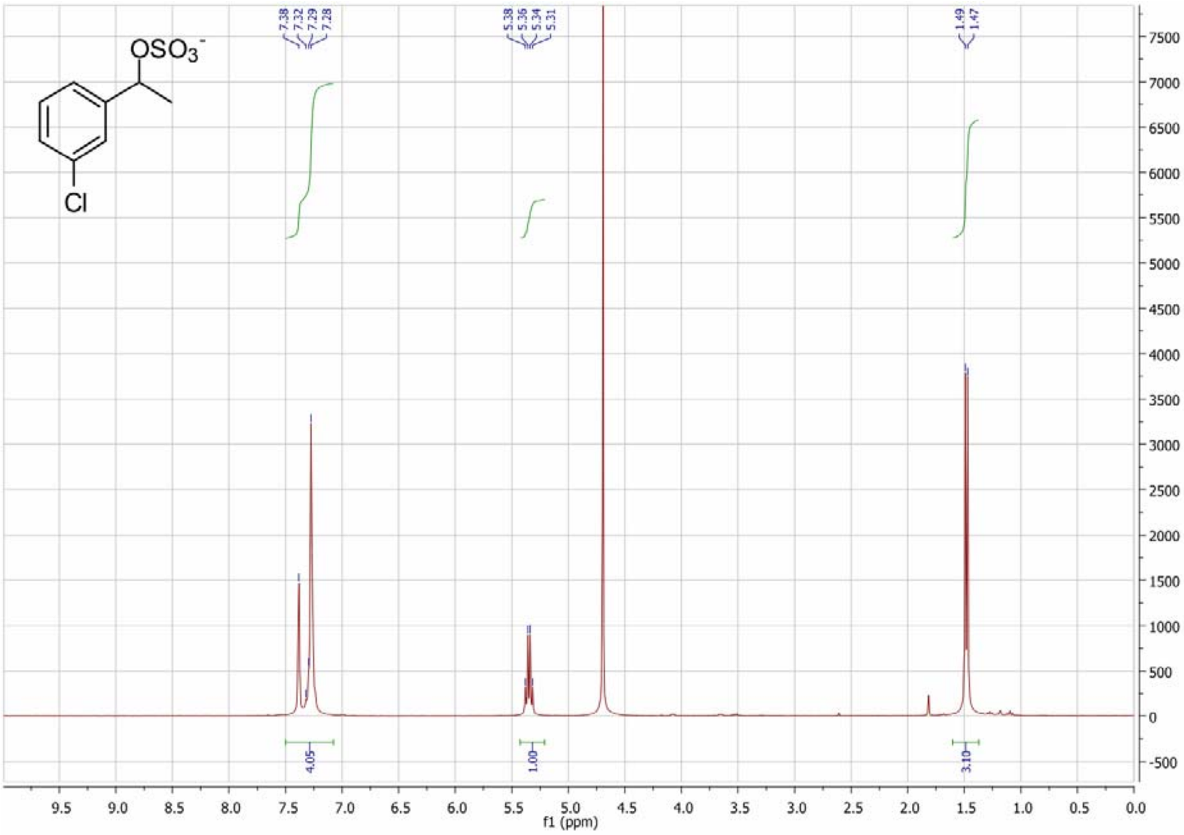

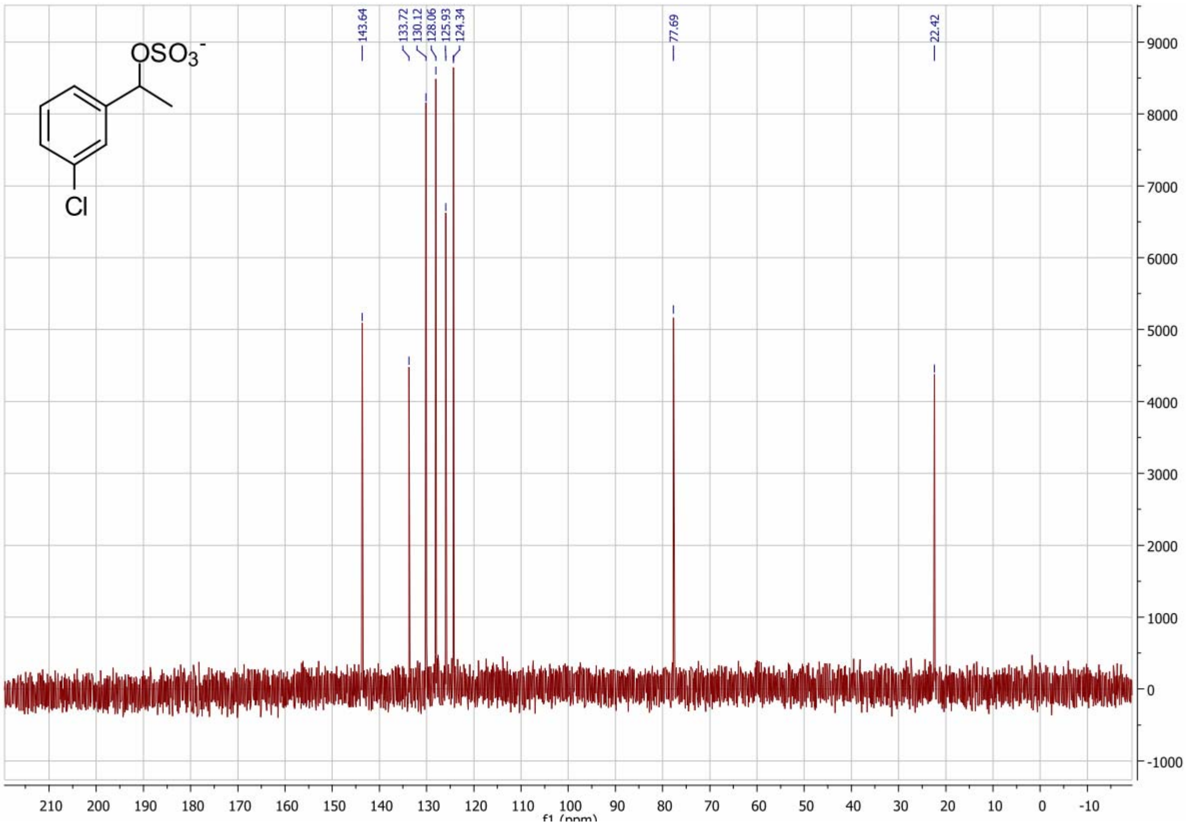

rac-[1-(3-Trifluoromethyl)phenyl]ethyl sulfate (**6a**) DMSO-d_6

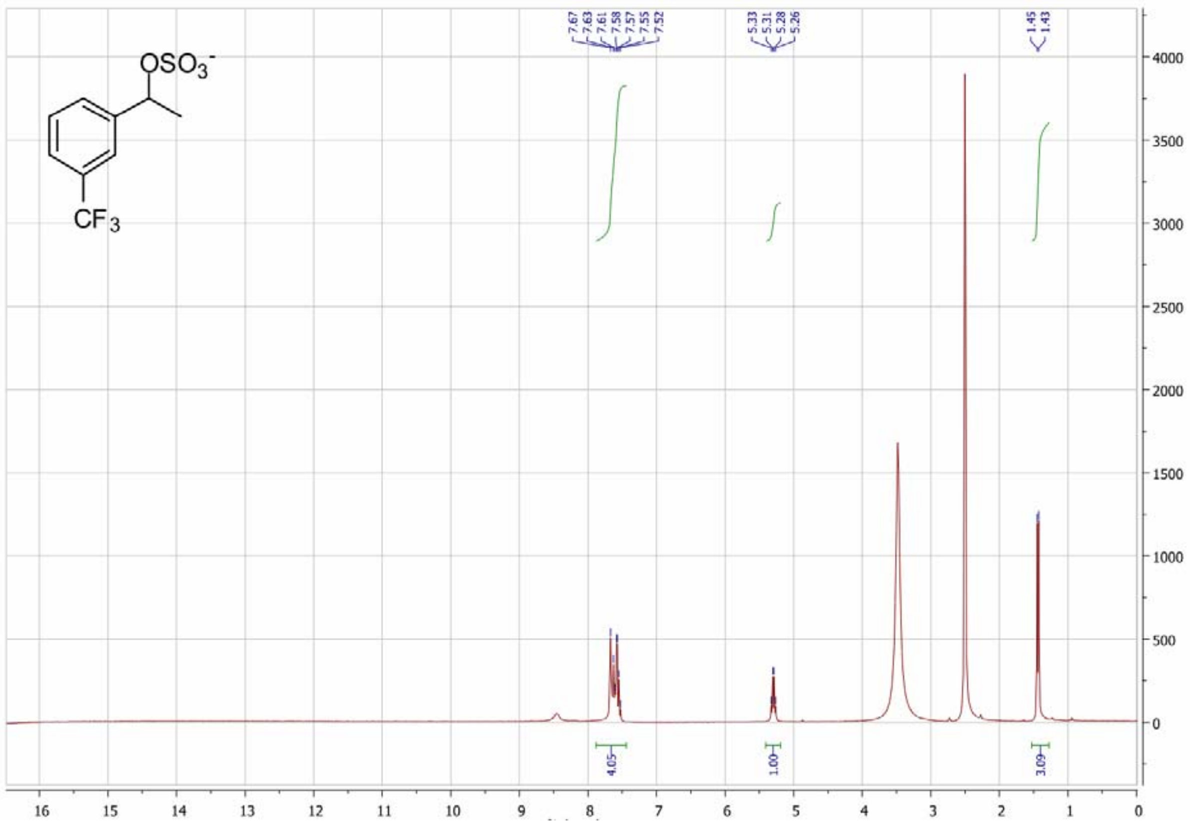

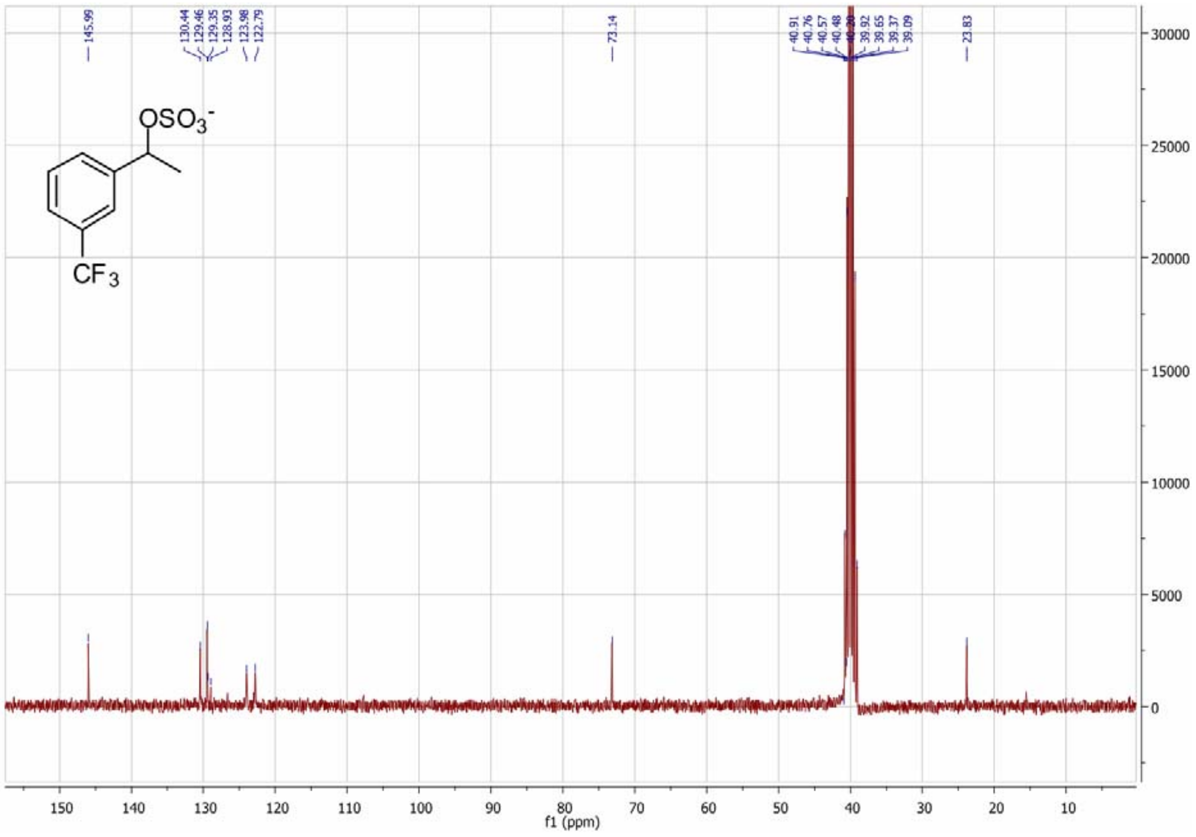

rac-1-(Pyridin-3-yl)ethyl sulfate (**8a**) D_2O

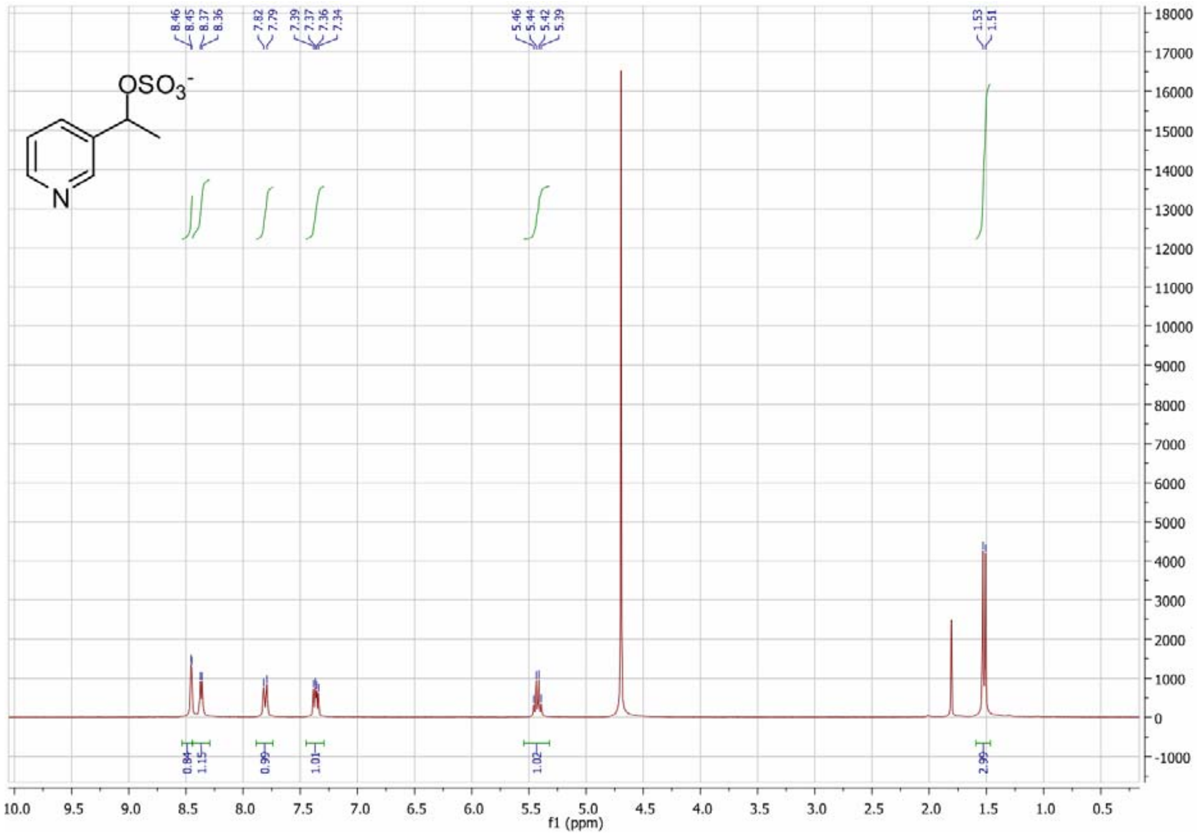

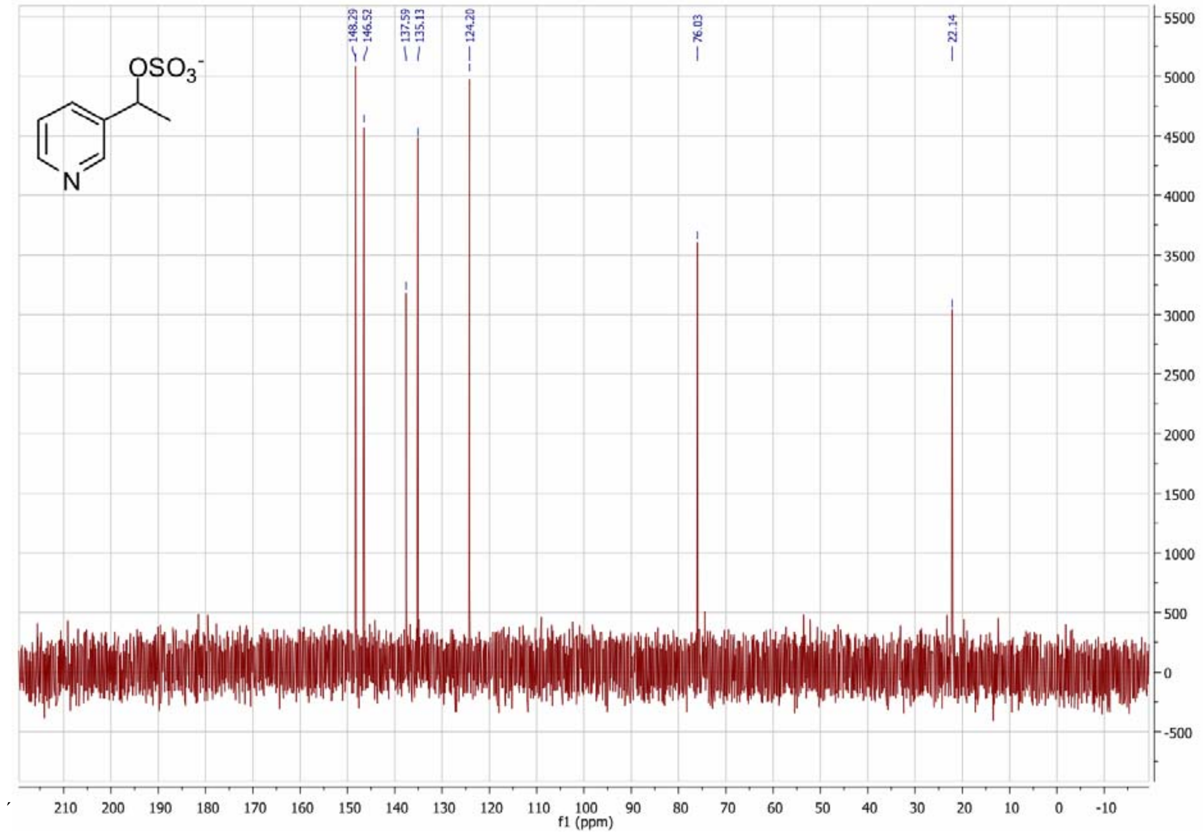

GC-MS Measurements and ^{18}O-labelling data

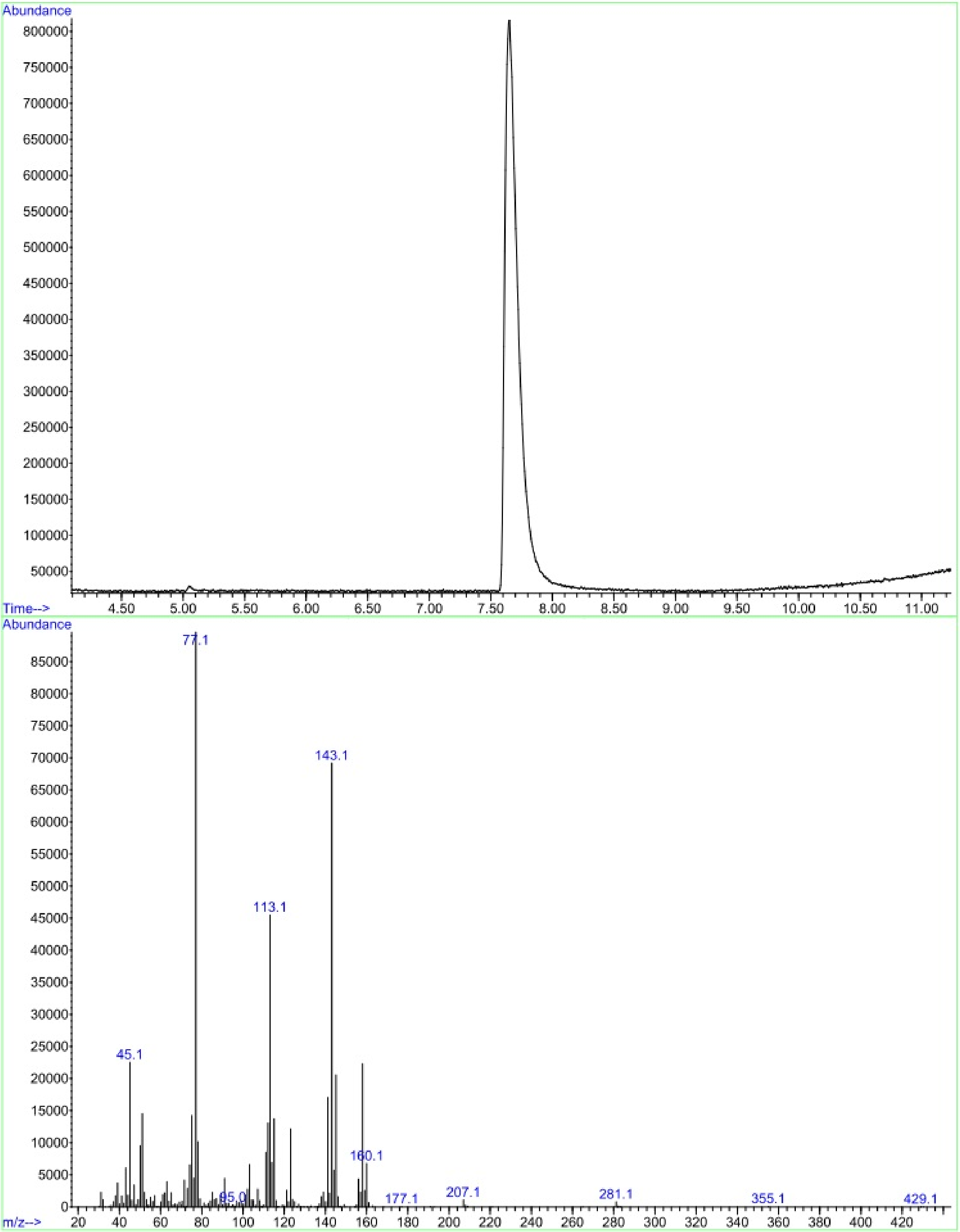

Figure S1: GC-MS of (*S*)-**5b** (e.r. >99:<1) from enzymatic hydrolysis of (*R*)-**5a** in ^{18}O-labeled H_2O.

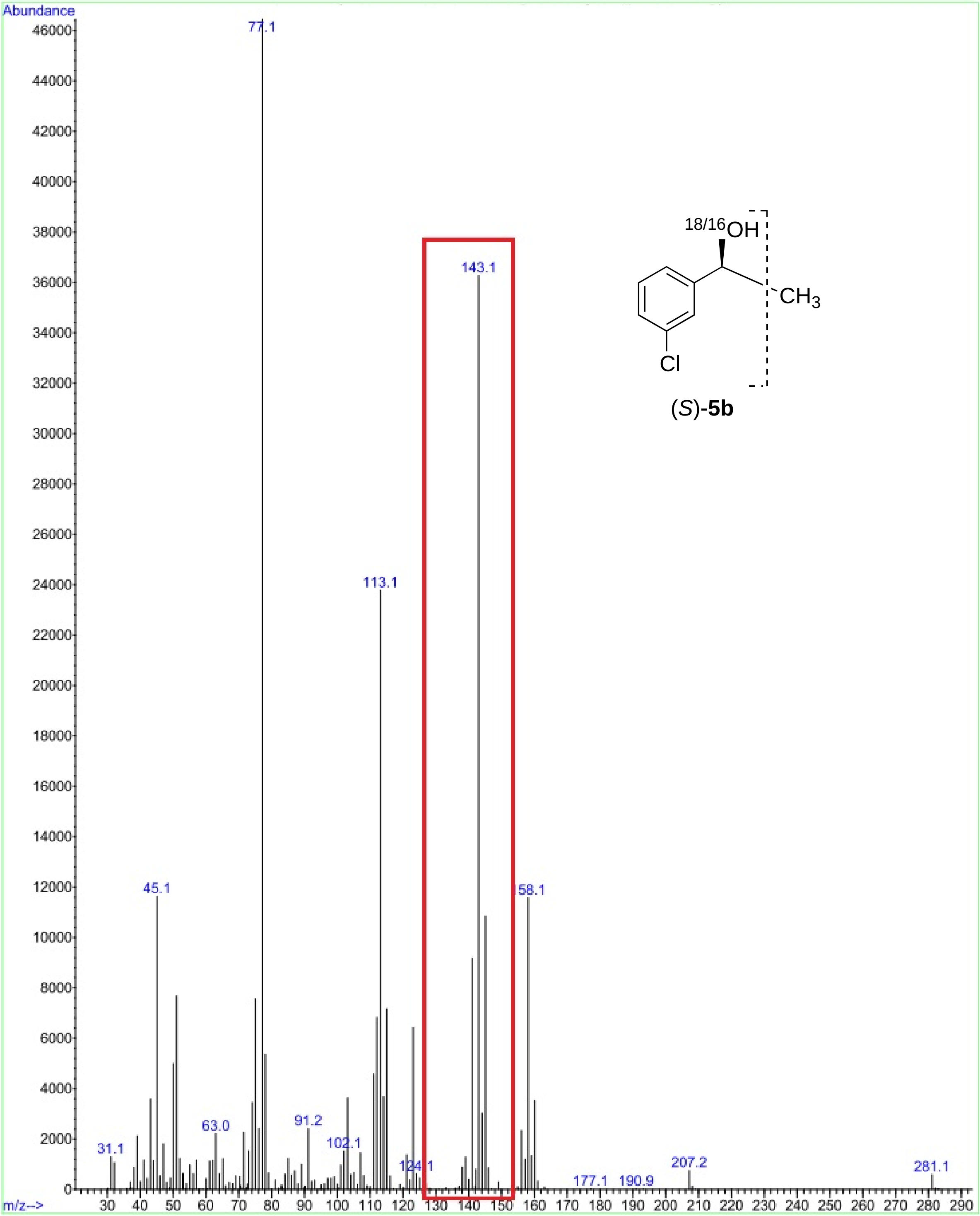

Figure S2: Blow-up of MS spectrum of (*S*)-**5b** (Fig. S1, bottom), the section containing the $^{18/16}$O-labeled fragments at 143/141 is highlighted.

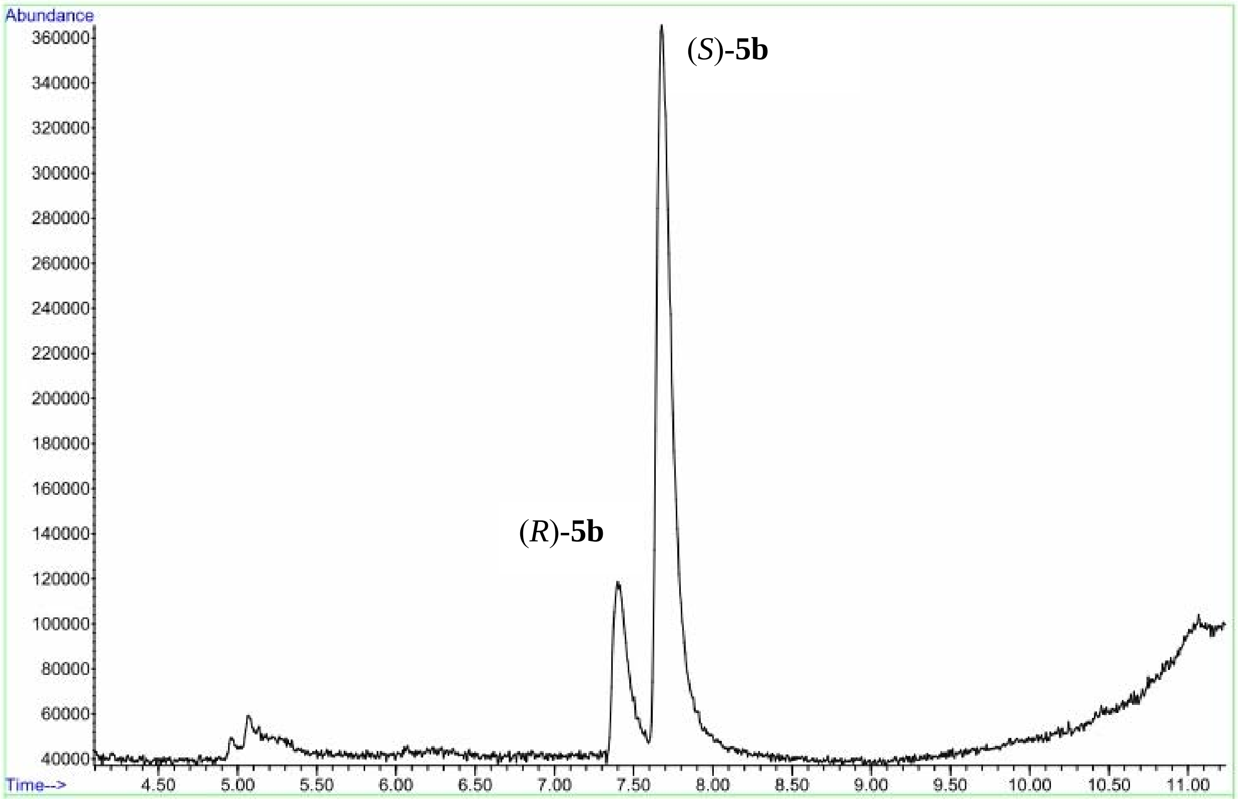

Figure S3: GC-MS of (*S*)-**5b** (e.r. 81:19) from autohydrolysis of (*R*)-**5a** in ^{18}O-labeled H_2O.

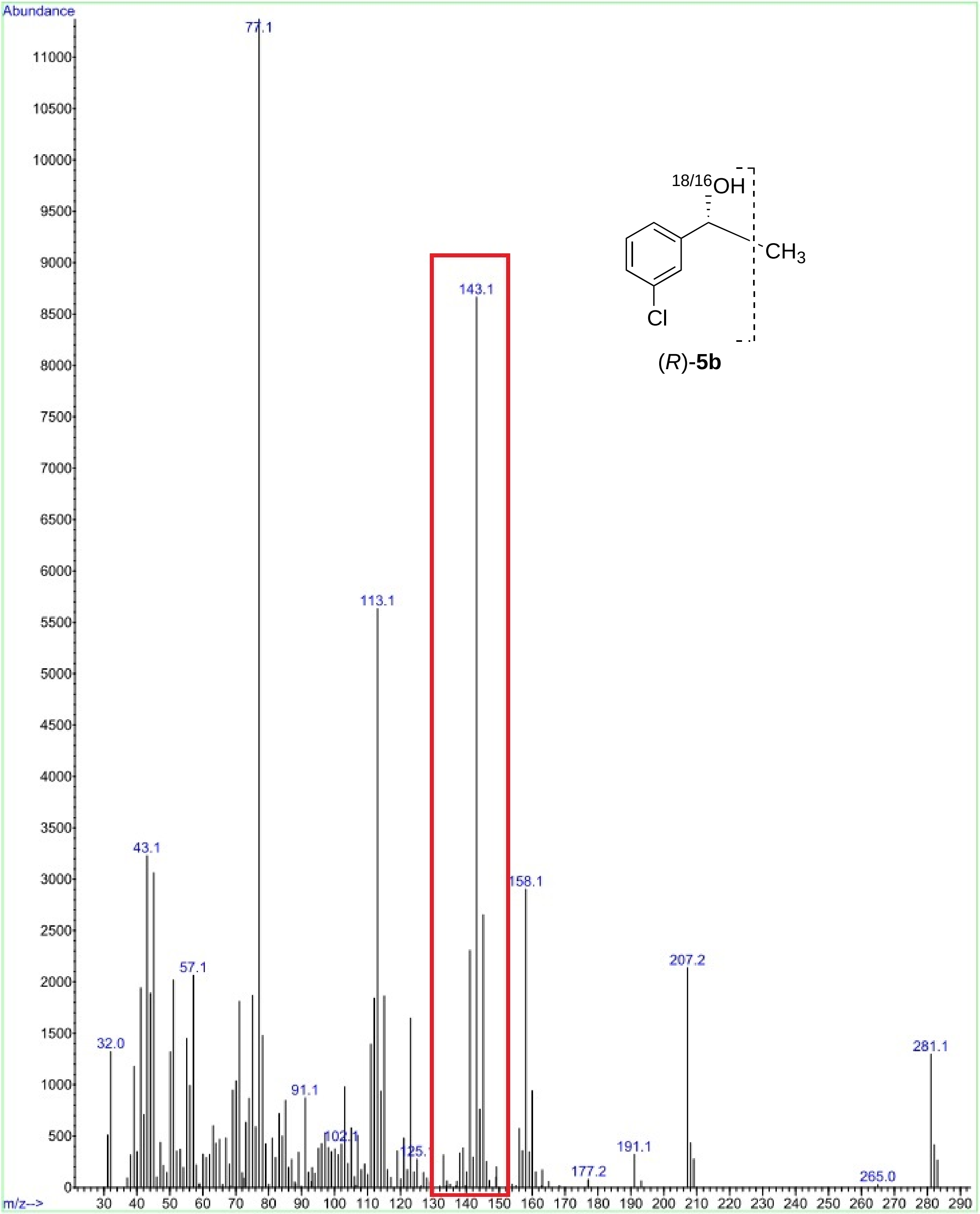

Figure S4: MS spectrum of peak for (*R*)-**5b** (Fig. S3), the section containing the $^{18/16}$O-labeled fragments at 143/141 is highlighted.

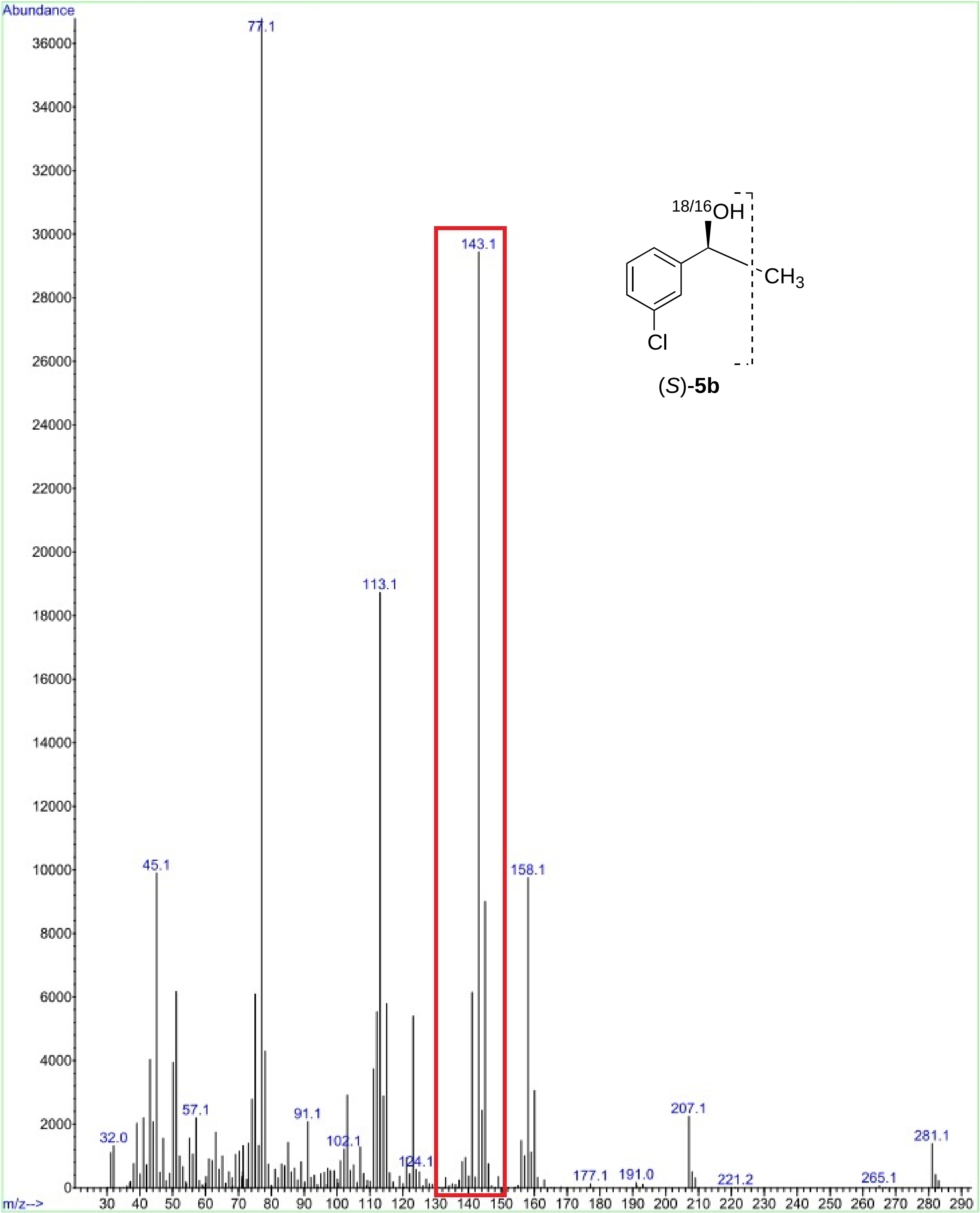

Figure S5: MS spectrum of peak for (*S*)-**5b** (Fig. S3), the section containing the $^{18/16}$O-labeled fragments at 143/141 is highlighted.

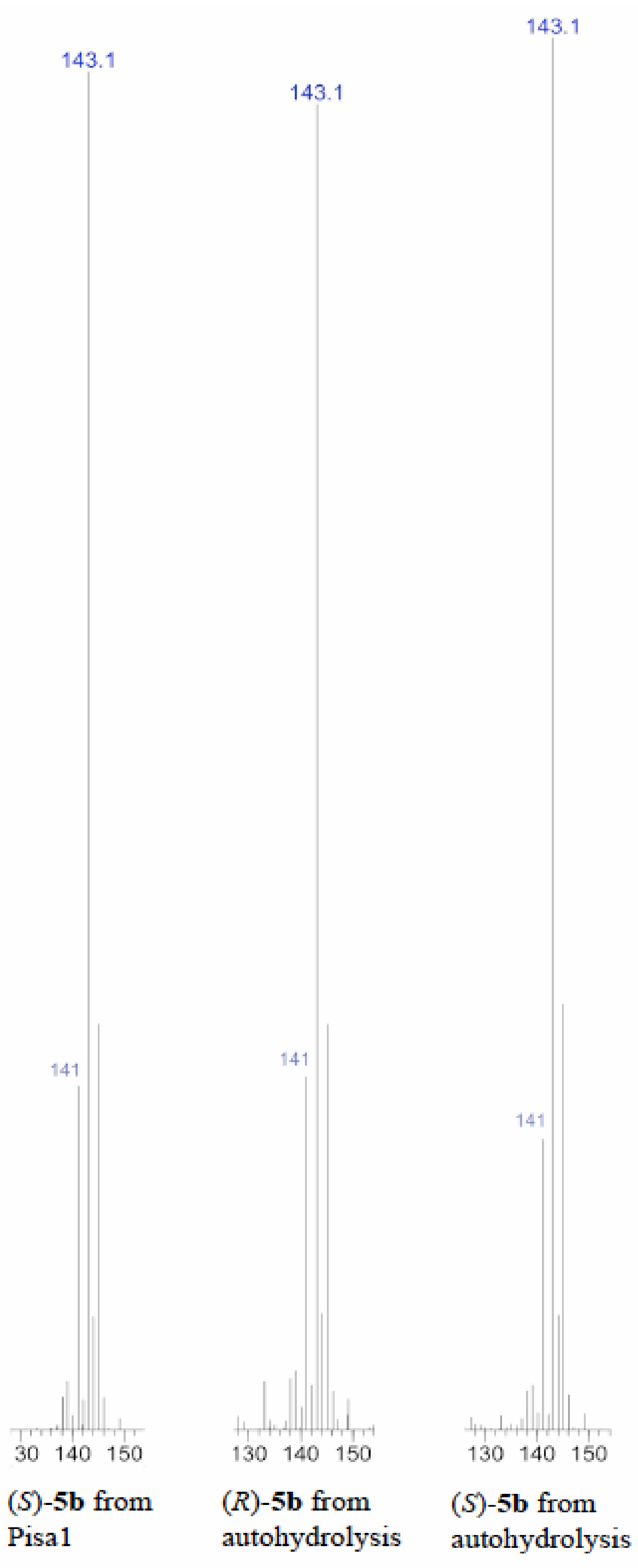

Figure S6: Blow-up of the highlighted sections from Figs. 2, 4 and 5.

References

[1] K. Edegger, C. C. Gruber, K. Faber, A. Hafner, W. Kroutil, *Eng. Life Sci.* **2006**, *6*, 149-154.

[2] M. Schober, P. Gadler, T. Knaus, H. Kayer, R. Birner-Grünberger, C. Gülly, P. Macheroux, U. Wagner, K. Faber, *Org. Lett.* **2011**, *13*, 4296-4299.

[3] M. Schober, T. Knaus, M. Toesch, P. Macheroux, U. Wagner, K. Faber, *Adv. Syn. Catal.* **2012**, *354*, 1737-1742.

[4] J.-H. Xie, X.-Y. Liu, J.-B. Xie, L.-X. Wang, Q.-L. Zhou, *Angew. Chem. Int. Ed.* **2011**, *50*, 7329-7332.

[5] N. A. Salvi, S. Chattopadhyay, *Tetrahedron: Asymmetry* **2008**, *19*, 1992-1997.

[6] T. Hayashi, S. Hirate, K. Kitayama, H. Tsuji, A. Torii, Y. Uozumi, *J. Org. Chem.* **2001**, *66*, 1441-1449.

[7] D. R. Li, A. He, J. R. Falck, *Org. Lett.* **2010**, *12*, 1756-1759.

[8] V. Aldabalde, P. Arcia, A. Gonzalez, D. Gonzalez, *Green Chem. Lett. Rev.* **2007**, *1*, 25-30.
